# Supplementary material for: Unveiling the Bioactive Potential of the Invasive Jellyfish Phyllorhiza punctata Through Integrative Transcriptomic and Proteomic Analyses
Source: Biomolecules. 2025 Aug 4;15(8):1121. doi: 10.3390/biom15081121 (PMC12383608; doi:10.3390/biom15081121)
Supplement: Supplementary file 1 [file biomolecules-15-01121-s001.zip › Suplementary Material/FileS3/PEPFOLD4/XP_065071437_6421/clusters.html]

 PEP-FOLD clusters


Download archive of all models  here   

|  |  |  |
| --- | --- | --- |
| # | file | sOPEP |
| # cluster 1 - Size: 3 |  |  |
|  | XP\_065071437\_6421-model1 | -3.53539 |
|  | XP\_065071437\_6421-model1.1 | -3.5204 |
|  | XP\_065071437\_6421-model1.2 | -3.50279 |
| # cluster 2 - Size: 3 |  |  |
|  | XP\_065071437\_6421-model2 | -3.53435 |
|  | XP\_065071437\_6421-model2.1 | -3.44036 |
|  | XP\_065071437\_6421-model2.2 | -3.41483 |
| # cluster 3 - Size: 17 |  |  |
|  | XP\_065071437\_6421-model3 | -3.52556 |
|  | XP\_065071437\_6421-model3.1 | -3.52556 |
|  | XP\_065071437\_6421-model3.2 | -3.5088 |
|  | XP\_065071437\_6421-model3.3 | -3.5088 |
|  | XP\_065071437\_6421-model3.4 | -3.50782 |
|  | XP\_065071437\_6421-model3.5 | -3.45335 |
|  | XP\_065071437\_6421-model3.6 | -3.45335 |
|  | XP\_065071437\_6421-model3.7 | -3.44639 |
|  | XP\_065071437\_6421-model3.8 | -3.44401 |
|  | XP\_065071437\_6421-model3.9 | -3.44002 |
|  | XP\_065071437\_6421-model3.10 | -3.4376 |
|  | XP\_065071437\_6421-model3.11 | -3.43013 |
|  | XP\_065071437\_6421-model3.12 | -3.43013 |
|  | XP\_065071437\_6421-model3.13 | -3.41511 |
|  | XP\_065071437\_6421-model3.14 | -3.39355 |
|  | XP\_065071437\_6421-model3.15 | -3.38016 |
|  | XP\_065071437\_6421-model3.16 | -3.36009 |
| # cluster 4 - Size: 22 |  |  |
|  | XP\_065071437\_6421-model4 | -3.52116 |
|  | XP\_065071437\_6421-model4.1 | -3.52092 |
|  | XP\_065071437\_6421-model4.2 | -3.47224 |
|  | XP\_065071437\_6421-model4.3 | -3.45589 |
|  | XP\_065071437\_6421-model4.4 | -3.41497 |
|  | XP\_065071437\_6421-model4.5 | -3.41497 |
|  | XP\_065071437\_6421-model4.6 | -3.41497 |
|  | XP\_065071437\_6421-model4.7 | -3.41 |
|  | XP\_065071437\_6421-model4.8 | -3.41 |
|  | XP\_065071437\_6421-model4.9 | -3.41 |
|  | XP\_065071437\_6421-model4.10 | -3.40797 |
|  | XP\_065071437\_6421-model4.11 | -3.40797 |
|  | XP\_065071437\_6421-model4.12 | -3.40772 |
|  | XP\_065071437\_6421-model4.13 | -3.40551 |
|  | XP\_065071437\_6421-model4.14 | -3.3987 |
|  | XP\_065071437\_6421-model4.15 | -3.39138 |
|  | XP\_065071437\_6421-model4.16 | -3.39138 |
|  | XP\_065071437\_6421-model4.17 | -3.38846 |
|  | XP\_065071437\_6421-model4.18 | -3.37636 |
|  | XP\_065071437\_6421-model4.19 | -3.37436 |
|  | XP\_065071437\_6421-model4.20 | -3.36741 |
|  | XP\_065071437\_6421-model4.21 | -3.36741 |
| # cluster 5 - Size: 2 |  |  |
|  | XP\_065071437\_6421-model5 | -3.49399 |
|  | XP\_065071437\_6421-model5.1 | -3.434 |
| # cluster 6 - Size: 9 |  |  |
|  | XP\_065071437\_6421-model6 | -3.48599 |
|  | XP\_065071437\_6421-model6.1 | -3.48599 |
|  | XP\_065071437\_6421-model6.2 | -3.45896 |
|  | XP\_065071437\_6421-model6.3 | -3.45896 |
|  | XP\_065071437\_6421-model6.4 | -3.45896 |
|  | XP\_065071437\_6421-model6.5 | -3.43075 |
|  | XP\_065071437\_6421-model6.6 | -3.37877 |
|  | XP\_065071437\_6421-model6.7 | -3.37227 |
|  | XP\_065071437\_6421-model6.8 | -3.35982 |
| # cluster 7 - Size: 8 |  |  |
|  | XP\_065071437\_6421-model7 | -3.48335 |
|  | XP\_065071437\_6421-model7.1 | -3.42971 |
|  | XP\_065071437\_6421-model7.2 | -3.42971 |
|  | XP\_065071437\_6421-model7.3 | -3.41499 |
|  | XP\_065071437\_6421-model7.4 | -3.41382 |
|  | XP\_065071437\_6421-model7.5 | -3.40977 |
|  | XP\_065071437\_6421-model7.6 | -3.38633 |
|  | XP\_065071437\_6421-model7.7 | -3.3638 |
| # cluster 8 - Size: 4 |  |  |
|  | XP\_065071437\_6421-model8 | -3.47853 |
|  | XP\_065071437\_6421-model8.1 | -3.44868 |
|  | XP\_065071437\_6421-model8.2 | -3.39908 |
|  | XP\_065071437\_6421-model8.3 | -3.38152 |
| # cluster 9 - Size: 6 |  |  |
|  | XP\_065071437\_6421-model9 | -3.47741 |
|  | XP\_065071437\_6421-model9.1 | -3.47087 |
|  | XP\_065071437\_6421-model9.2 | -3.4704 |
|  | XP\_065071437\_6421-model9.3 | -3.45983 |
|  | XP\_065071437\_6421-model9.4 | -3.44376 |
|  | XP\_065071437\_6421-model9.5 | -3.35925 |
| # cluster 10 - Size: 11 |  |  |
|  | XP\_065071437\_6421-model10 | -3.47701 |
|  | XP\_065071437\_6421-model10.1 | -3.4727 |
|  | XP\_065071437\_6421-model10.2 | -3.4727 |
|  | XP\_065071437\_6421-model10.3 | -3.46164 |
|  | XP\_065071437\_6421-model10.4 | -3.45885 |
|  | XP\_065071437\_6421-model10.5 | -3.43591 |
|  | XP\_065071437\_6421-model10.6 | -3.43217 |
|  | XP\_065071437\_6421-model10.7 | -3.43206 |
|  | XP\_065071437\_6421-model10.8 | -3.41915 |
|  | XP\_065071437\_6421-model10.9 | -3.40495 |
|  | XP\_065071437\_6421-model10.10 | -3.36253 |
| # cluster 11 - Size: 1 |  |  |
|  | XP\_065071437\_6421-model11 | -3.45325 |
| # cluster 12 - Size: 2 |  |  |
|  | XP\_065071437\_6421-model12 | -3.45097 |
|  | XP\_065071437\_6421-model12.1 | -3.42258 |
| # cluster 13 - Size: 1 |  |  |
|  | XP\_065071437\_6421-model13 | -3.43415 |
| # cluster 14 - Size: 4 |  |  |
|  | XP\_065071437\_6421-model14 | -3.41794 |
|  | XP\_065071437\_6421-model14.1 | -3.41498 |
|  | XP\_065071437\_6421-model14.2 | -3.39993 |
|  | XP\_065071437\_6421-model14.3 | -3.38908 |
| # cluster 15 - Size: 5 |  |  |
|  | XP\_065071437\_6421-model15 | -3.41159 |
|  | XP\_065071437\_6421-model15.1 | -3.40781 |
|  | XP\_065071437\_6421-model15.2 | -3.40165 |
|  | XP\_065071437\_6421-model15.3 | -3.37952 |
|  | XP\_065071437\_6421-model15.4 | -3.37952 |
| # cluster 16 - Size: 1 |  |  |
|  | XP\_065071437\_6421-model16 | -3.39625 |
| # cluster 17 - Size: 1 |  |  |
|  | XP\_065071437\_6421-model17 | -3.3815 |
|  |  |  |
